# Quantitative chest computed tomography predicts mortality in systemic sclerosis: A longitudinal study

**DOI:** 10.1371/journal.pone.0310892

**Published:** 2024-09-27

**Authors:** Fernanda Godinho Amorim, Ernandez Rodrigues dos Santos, Carlos Gustavo Yuji Verrastro, Cristiane Kayser

**Affiliations:** 1 Rheumatology Division, Escola Paulista de Medicina, Universidade Federal de São Paulo (UNIFESP), São Paulo, Brazil; 2 Department of Radiology, Escola Paulista de Medicina, Universidade Federal de São Paulo (UNIFESP), São Paulo, Brazil; Nippon Medical School, JAPAN

## Abstract

**Objective:**

Quantitative chest computed tomography (qCT) methods are new tools that objectively measure parenchymal abnormalities and vascular features on CT images in patients with interstitial lung disease (ILD). We aimed to investigate whether the qCT measures are predictors of 5-year mortality in patients with systemic sclerosis (SSc).

**Methods:**

Patients diagnosed with SSc were retrospectively selected from 2011 to 2022. Patients should have had volumetric high-resolution CTs (HRCTs) and pulmonary function tests (PFTs) performed at baseline and at 24 months of follow-up. The following parameters were evaluated in HRCTs using Computer-Aided Lung Informatics for Pathology Evaluation and Rating (CALIPER): ground glass opacities, reticular pattern, honeycombing, and pulmonary vascular volume. Factors associated with death were evaluated by Kaplan‒Meier survival curves and multivariate analysis models. Semiquantitative analysis of the HRCTs images was also performed.

**Results:**

Seventy-one patients were included (mean age, 54.2 years). Eleven patients (15.49%) died during the follow-up, and all patients had ILD. As shown by Kaplan‒Meier curves, survival was worse among patients with an ILD extent (ground glass opacities + reticular pattern + honeycombing) ≥ 6.32%, a reticular pattern ≥ 1.41% and a forced vital capacity (FVC) < 70% at baseline. The independent predictors of mortality by multivariate analysis were a higher reticular pattern (Exp 2.70, 95%CI 1.26–5.82) on qCT at baseline, younger age (Exp 0.906, 95%CI 0.826–0.995), and absolute FVC decline ≥ 5% at follow-up (Exp 15.01, 95%CI 1.90–118.5), but not baseline FVC. Patients with extensive disease (>20% extension) by semiquantitative analysis according to Goh’s staging system had higher disease extension on qCT at baseline and follow-up.

**Conclusion:**

This study showed that the reticular pattern assessed by baseline qCT may be a useful tool in the clinical practice for assessing lung damage and predicting mortality in SSc.

## Introduction

Systemic sclerosis (SSc) is a heterogeneous autoimmune rheumatic disease characterized by vascular abnormalities, immune activation, and fibrosis of the skin and internal organs [1]. Interstitial lung disease (ILD) is a frequent manifestation in patients with SSc and is currently the leading cause of death in this population [2].

Estimates of the prevalence of ILD in patients with SSc vary from 35% to 80% depending on the population and the criteria or method used to define it [3]. Early diagnosis of ILD is important to allow more effective management and a better prognosis. Nonetheless, the disease course in individual patients and the patients who will need early treatment are often difficult to predict [4–7].

Chest high-resolution computed tomography (HRCT) and pulmonary function tests (PFTs) are the current methods used for the diagnosis and monitoring of SSc-ILD. HRCT is the gold standard method for early detection of ILD and is also useful for evaluating disease extent and progression in patients with SSc [8–10]. Seminal studies have shown a worse prognosis in patients with a greater disease extent on semiquantitative assessment on HRCT and a forced vital capacity (FVC) < 70% [11]. Moreover, a serial decline in FVC is also strongly associated with a higher risk of mortality [12]. Nonetheless, both methods present limitations such as low reproducibility and low sensitivity. Conventional CT image analysis has high inter- and intraobserver variability and low efficiency in recognizing subtle changes during follow-up [13, 14]. It has also been considered to have low applicability to treatment response evaluations in therapeutic clinical trials due to the volume of images and the time required to evaluate the sections [14]. Changes in FVC and the carbon monoxide diffusing capacity of the lungs (DLco) have been widely used to assess disease progression and the response to therapy in clinical trials [15, 16]. However, previous studies have shown a lack of sensitivity of PFTs for the detection of ILD in general SSc cohorts, especially in patients with early stages of the disease [17, 18]. Variability determined by technical factors and diurnal or seasonal changes, among others, can also affect the reliability of FVC results [19].

With the advent of new technologies and in response to the limitations of current methods, effort has been made to develop more reproducible and sensitive imaging techniques for the detection, quantification, and better characterization of ILD [20]. One of these methods is computer-aided tomographic quantification (qCT), such as Computer-Aided Lung Informatics for Pathology Evaluation and Rating (CALIPER), which employs a technique based on texture analysis of the lung parenchyma [21–25]. Although widely explored in idiopathic pulmonary fibrosis (IPF) [24–29], few studies have evaluated this technology in lung disease associated with SSc [30–32].

Therefore, the main objective of this study was to evaluate quantitative lung assessment using CALIPER at baseline and during a follow-up of 24 months as a predictor of mortality in 5 years in patients with SSc. Furthermore, we investigated correlations between CALIPER parameters and PFTs, the association between CALIPER and semiquantitative HRCT analysis and the capacity of qCT to measure disease progression during follow-up.

## Methods

### Study design and patient population

This was a retrospective longitudinal single-center cohort study evaluating patients with SSc according to the 2013 ACR/EULAR criteria [33] who were followed up in the Scleroderma Outpatient Clinic at Federal University of São Paulo’s Medical School Hospital. Patients over 18 years of age, seen from March 26, 2011 to October 24, 2022, were contacted by telephone and invited to participate in the research. Patients who agreed to participate signed a form detailing the research and allowing the use of data in the study. Data security and images were anonymized. In general terms, data identifiers were removed and replaced with a single key code through the sole and exclusive possession of the main researcher and all data were deleted at the end of the study.

The inclusion criteria were as follows: (1) patients older than 18 years and (2) patients with two HRCT evaluations and two PFTs performed with an interval of 24 months between the first and the second exams. In addition, the PFTs needed to be performed with an interval no longer than 3 months after HRCT. Patients with a history of smoking, suspected infection close to the date of testing and low-quality HCRT results or who did not meet image acquisition were excluded. The participants had the following clinical and demographic variables of interest extracted from medical records: age, sex, disease duration (defined as the time between the first non-Raynaud’s symptom and the last available evaluation), disease subtype (limited or diffuse cutaneous SSc), digital ulcers, renal crisis and autoantibodies (antinuclear antibody screening by indirect immunofluorescence on HEp-2 cells, anti-topoisomerase I and anti-centromere antibodies). The presence of pulmonary arterial hypertension (PAH) was confirmed by right heart catheterization (RHC) and defined as a mean pulmonary arterial pressure (mPAP) ≥20 mmHg with a pulmonary artery wedge pressure (PAWP) ≤15 mmHg and pulmonary vascular resistance (PVR) > 3 Wood units [34]. Data on the use of immunosuppressive therapy were also collected.

Death and its causes were identified in electronic medical records or by contact with family members and reported if related to SSc.

This study was carried out in accordance with the Declaration of Helsinki, and all patients signed written informed consent. The study was approved by the Local Ethics Committee (CEP/UNIFESP n: 0288/2021).

### Pulmonary function testing

The functional lung assessment, particularly FVC and DLco evaluations, was performed according to the American Thoracic Society/European Respiratory Society (ATS/ERS) guidelines [35, 36] using standard equipment (Elite Series TM Plethysmograph; MedGraphics Cardiorespiratory Diagnostic Systems—Medical Graphics Corporation Inc., 2005, St Paul, MN, USA). The results were expressed as percentages of predicted (% pred) values. Absolute declines in the FVC of ≥ 10% and of ≥ 5% (% pred) over 24 months were also analyzed.

### High-resolution computed tomography

HRCT scans were obtained with a Phillips Brilliance 64-slice scanner (Phillips Medical Systems Inc., 2005, Cleveland, USA). Acquisition parameters were set to 120 kVp, 212 mAs, rotation time 0.5 s, pitch 1.0, and 1-mm-thick axial slices, without contrast, and were obtained with the patient in the dorsal decubitus position at full inspiration. Images were reconstructed using an image matrix with 512x512 pixels and a B kernel.

The presence of ILD on HRCT scans was defined by the presence of parenchymal abnormalities greater than 5% as evaluated by two rheumatologists with expertise in SSc.

### Visual semiquantitative assessment of ILD extent

We used the visual assessment of total lung involvement proposed by Goh and Wells. HRCT images were scored by two independent rheumatologists at five levels, and the HRCT disease extent threshold of 20% was used to stratify ILD extent as < 20% and > 20% (limited versus extensive). The FVC threshold of 70% was used in indeterminate cases [11].

### Tomographic quantification (qCT)

**Fig 1:**
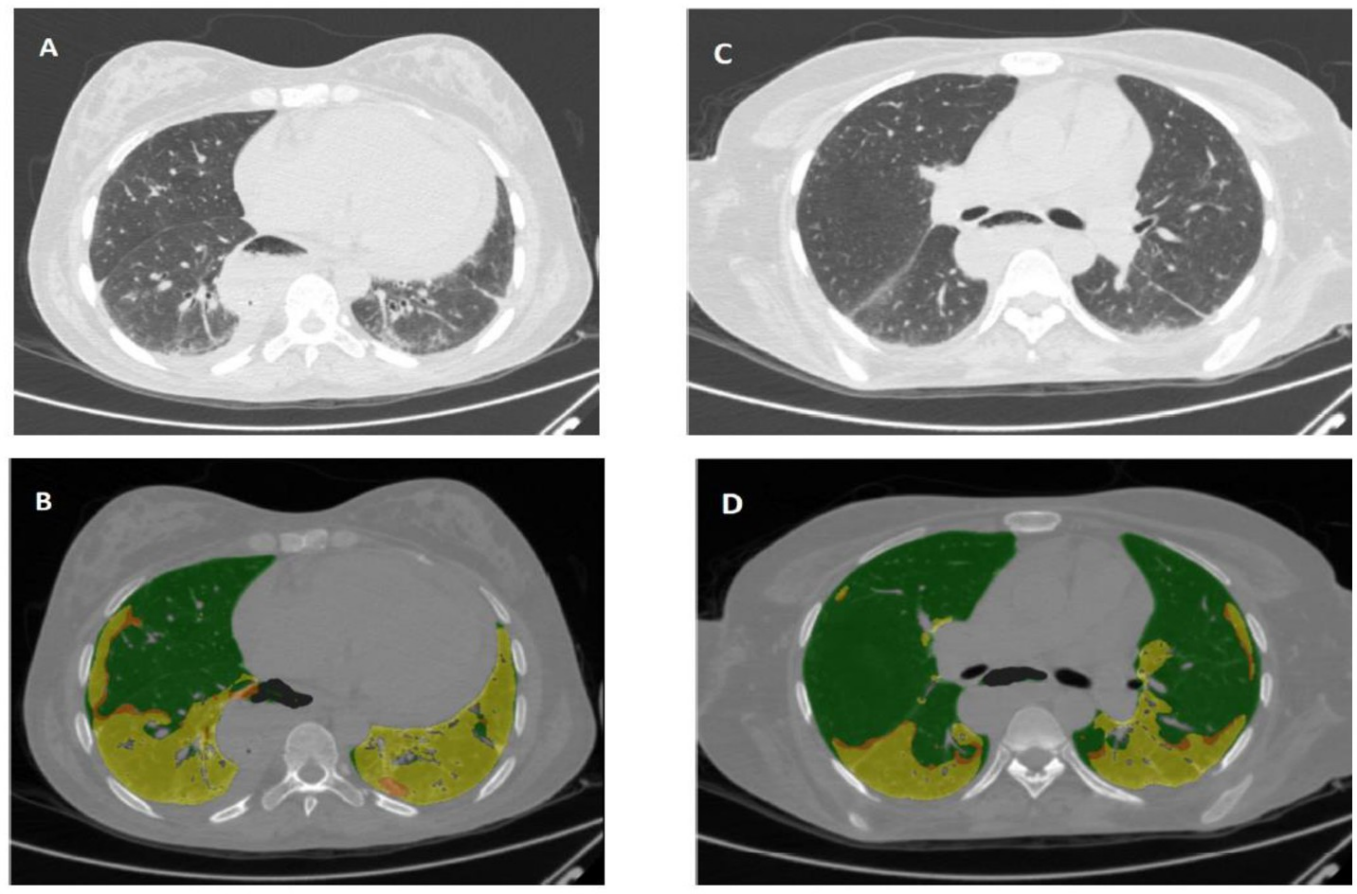
Illustrative image of the CALIPER of a patient with SSc and ILD. Images were taken at time 0 (Fig 1A and 1B) and 24 months (Fig 1C and 1D). The individual voxels of lung regions in the original sections (A and C) are sorted and color-coded into one of the classes of visible abnormalities (B and D). (Image from the author’s personal archive).

Quantitative analyses were performed automatically using Lung Texture Analysis software, IMBIO version 22.0, based on CALIPER technology (Biomedical Imaging Resource. Mayo Clinic Rochester, MN, USA) (Fig 1). The software analyzes the presence of specific abnormalities and their percentage distribution per lung segment into six patterns: ground-glass opacity, reticular pattern, honeycombing, normal lung, total lung volume (LV) and pulmonary vessel volume (PVV) as previously described [20–22]. The detection and quantification of each pulmonary parenchyma pattern uses different metrics based on histogram signature mapping techniques descriptors that were tested for their ability to discriminate among the six categories [22].

The measurements were given in total volume in cm3 and the overall lung percentage. In addition, we evaluated the extent of interstitial lung disease (ILD extent% = ground glass, reticulate and total honeycombing in percentage), and the fibrosis score (%) as the sum of reticulate and total honeycombing as a percentage, as well as the vascular index (%) given by PVV over LV [23, 25].

### Statistical analysis

Clinical and demographic data were described using frequencies and percentages for qualitative variables and the mean and standard deviation or the median and interquartile range (IQR) for quantitative variables. The Kolmogorov‒Smirnov test was used to evaluate the normality distribution. The Wilcoxon test was used to compare continuous variables between groups throughout the follow-up. Correlations between lung quantification software measurements and PFT variables were assessed using the Spearman correlation test.

Receiver operating characteristic (ROC) curves were used to estimate the diagnostic accuracy of the parameters generated by CALIPER at baseline and follow-up to predict mortality. The best cutoff values for each variable were calculated based on Youden’s index criteria (S1 Table).

Kaplan‒Meier curves with log-rank tests were used for comparisons of overall survival between groups. Survival was censored at 5 years from the first HRCT and PFTs evaluation. Mortality rates at 1, 3 and 5 years were calculated. In addition, presumed risk factors associated with death were assessed using a univariate Cox regression model. The time to event (death) or censorship was measured from the first dataset (HRCT and PFTs at baseline).

A multivariate analysis of clinical, functional and pulmonary quantification parameters’ abilities to predict death was performed using generalized linear models (GLMs), and the best model was determined using the p value and the Akaike information criterion (AIC). Hazard ratios (HRs) with 95% confidence intervals (CIs) were calculated.

For all analyses, a value of p < 0.05 was considered statistically significant. Analyses were performed using SPSS statistical software (version 26.0 for Windows, SPSS Inc., Chicago, IL, USA). Survival curves were generated using GraphPad (GraphPad Software. LLC. 225 Boston. MA. USA).

## Results

### Demographic and clinical characteristics at baseline

Two hundred and twenty-three patients with SSc were identified. Of these, 197 had two serial HRCT scans with an interval of 24 months between them, and 78 had both two HRCT scans and two PFTs performed with an interval of 24 months between them and with a maximum interval of 3 months between each HRCT scan and PFTs. Seven patients were excluded because they did not meet the predefinitions of the lung quantification software. Thus, 71 patients were eligible for the study. Mostly were women (90.1%), with a mean age of 54.2 ± 11.6 years and a mean disease duration of 11 years. Sixty (84.5%) patients had ILD on HRCT and 32 patients (45%) had FVC <70%, pred. Table 1 describes the main clinical and demographic characteristics of the studied population.

**Table 1:** Clinical characteristics of SSc patients according to the presence or absence of interstitial lung disease (ILD) at baseline.

	Total (n = 71)	Patients with ILD (n = 60)
Age (mean ± SD), years	54.2 ± 11.6	53.4 ± 11.6
Female sex, n (%)	64 (90.1%)	54 (90%)
RP, n (%)	71 (100%)	60 (100%)
RP duration (mean ± SD), years	16.71 ± 9.77	14.41 ± 9.89
Disease duration, median (IQ), years	11.4 (7.00–14.00)	10 (7.00–14.00)
Cutaneous subset, n (%)		
Diffuse	26 (36.6%)	26 (43.3%)
Limited	45 (63.4%)	34 (56.7%)
FVC < 70%, n (%)	32 (45%)	30 (50%)
ILD, n (%)	60 (84.5%)	60 (100%)
Pulmonary arterial hypertension, n (%)	5 (7.04%)	3 (4.22%)
Digital ulcers, n (%)	8 (11.6%)	6 (10%)
Renal crisis, n (%)	0 (0%)	0 (0%)
Antinuclear antibodies, n (%)	67 (97.1%)	58 (98.3%)
Anti-topoisomerase I antibodies, n (%)	20 (31.3%)	19 (35.2%)
Anticentromere antibodies, n (%)	13 (22.4%)	10 (20.8%)
Death and main reason, n (%)	11 (15.5%)	11 (16.7%)
ILD, n (%)	5 (45.5%)	5 (45.5%)
Infection, n (%)	4 (36.4%)	4 (36.4%)
Malignancy, n (%)	2 (18.1%)	2 (18.1%)
Immunosuppressive treatment, n (%)	42 (59.2%)	39 (65%)
CYC/MMF/AZA/MTX/RTX	9 (21.4%)/7 (16.4%)/13 (31%)/6 (14%)/3 (7.1%)	7 (17.9%)/7(17.9%)/13 (33.3%)/6(15.4%)/2(5.1%)

AZA: azathioprine, CYC: cyclophosphamide, IQ: interquartile, ILD: interstitial lung disease, MMF: mycophenolate mofetil or sodium, MTX: methotrexate, RP: Raynaud’s phenomenon, RTX: rituximab

### PFTs and qCT at baseline and follow-up

The mean baseline values were 73.6% ± 19.1% for FVC and 54.3% ± 13.7% for the predicted DLco. At 24 months of follow-up, patients presented a significant absolute decline in FVC of -3.93 ± 8.92% pred (p = 0.001). A small decrease in lung volume was also observed -18.2 [–229–132], although not statistically significant (p = 0.278) (Table 2).

**Table 2:** Pulmonary function test (PFT) and pulmonary parameters quantified (qCT) by CALIPER at baseline and at 24 months of follow-up.

	BASELINE (n = 71)	FOLLOW-UP (n = 71)	Difference between T1 and T0	p
PFT (mean± SD)				
FVC, % pred	73.6 ± 19.1	69.6 ± 18.9	-3.93 ± 8.92	0.001
DLco, % pred	54.3 ± 13.7 (n = 8)	51.8 ± 13.9 (n = 9)	0.451± 20.2	0.696
CALIPER (median [IQ])				
Lung Volume, cm^3^	3345 [2792–4010]	3297 [2788–3906]	-18.2 [–229–132]	0.278
Normal, %	89.4 [79.1–95.5]	88.3 [75.4–94.8]	-0.51 [-4.92–0.87]	0.052
Fibrosis score, %	1.65 [0.68–3.25]	1.51 [0.67–3.19]	0.03 [-0.62–0.49]	0.920
ILD extent, %	7.82 [3.27–19.1]	7.96 [2.58–19.7]	0.150 [-2.05–2.84]	0.565
Ground glass opacities, %	6.87 [1.81–15.8]	6.55 [1.67–19.5]	0.128 [-2.75–2.76]	0.408
Reticular pattern, %	1.58 [0.67–2.85]	1.43 [0.66–3.19]	0.016 [-0.61–0.37]	0.720
Honeycombing, %	0.015 [0.01–0.05]	0.02 [0.005–0.04]	0.002 [0.00–0.03]	0.149
PVV, cm^3^	95.9 [75.9–128]	93.5 [71.8–136]	0.52 [-6.79–12.8]	0.334
PVV/LV, %	2.81 [2.09–4.20]	2.92 [2.00–4.45]	0.07 [-0.20–0.52]	0.143

DLco: carbon monoxide diffusing capacity, FVC: forced vital capacity, PVV: pulmonary vessel volume, PVV/LV: pulmonary vessel volume per lung volume

The qCT analyses showed a median extent of normal lung of 89.4% [IQR 79.1–95.5] at baseline. The median baseline extent of fibrosis was 1.65% [IQR 0.68–3.25], and the median extent of ILD was 7.82% [IQR 3.27–19.1]. Ground glass opacities were the most prominent parenchymal abnormalities, with an extent of involvement of 6.87% [IQR 1.81–15.8], followed by the reticular pattern with a median extent of 1.58% [IQR 0.671–2.85]. No significant variation in the quantified variables was noted between baseline and 24 months of follow-up (Table 2).

As expected, patients with an FVC < 70% pred at baseline had increased ILD involvement on qCT evaluation at baseline and follow-up compared to patients with mild functional impairment (FVC ≥ 70%, pred) (S2 Table).

We observed significant correlations between FVC and all patterns explored by qCT at baseline and follow-up, except for honeycombing. A significant correlation was also identified between DLco and most of the patterns quantified on qCT (S3 Table).

When comparing parameters quantified using CALIPER between those patients with extensive or limited disease (>20% versus < 20% extent on HCRT), patients with extensive disease had higher extents of disease quantified using CALIPER and lower FVC % at baseline and 24 months of follow-up (Table 3).

**Table 3:** Forced vital capacity (FVC) and pulmonary parameters quantified (qCT) by CALIPER according to < 20% or > 20% of ILD extent using the Goh’s staging system.

	< 20%extent	> 20% extent	P
**Baseline**			
FVC,%	83 (72–95)	63 (52–75.5)	< 0.001
ILD-extent%	3.12 (0.82–5.17)	17.6 (13–26)	< 0.001
Fibrosis score,%	0.62 (0.31–1.45)	2.74 (1.74–4.74)	< 0.001
Groundglass,%	1.63 (0.48–3.93)	15.4 (9.49–22.9)	< 0.001
Reticular,%	0.61 (0.31–1.44)	2.48 (1.69–4.70)	< 0.001
**Follow up**			
FVC,%	78 (71–91.3)	61 (54–69)	< 0.001
ILD-extent%	2.80 (1.07–5.59)	19 (11.6–34.8)	< 0.001
Fibrosis score,%	0.71 (0.29–1.12)	3.14 (1.81–4.63)	< 0.001
Groundglass,%	1.76 (0.73–4.71)	13.7 (9.61–28.4)	< 0.001
Reticular,%	0.70 (0.28–1.)	3.14 (1.80–4.26)	< 0.001

NOTE: ILD: interstitial lung disease, ILD extent, % = Ground-glass opacities, %+ reticular pattern, %+ Honeycombing, %.

### qCT and prognosis

Eleven patients (15.49%) died during mean follow-up times from diagnosis of 6.5 ± 1.97 years and from the first CT of 2.9 ± 0.83 years, including 9 (81.81%) women and 2 (18.18%) men, all of whom had ILD. ILD was the most frequent cause of death, accounting for 45.5% (5 patients) of all deaths, followed by infection in four patients (36.4%). The clinical, qCT and variables from the PFTs among the patients who died and those who survived are described in S4 Table. Interestingly, the mean absolute declines in FVC at 24 months of follow-up were -10.4 ± 10.4% pred in the group of patients who died and -2.75 ± 8.22% pred among those who remained alive.

**Fig 2:**
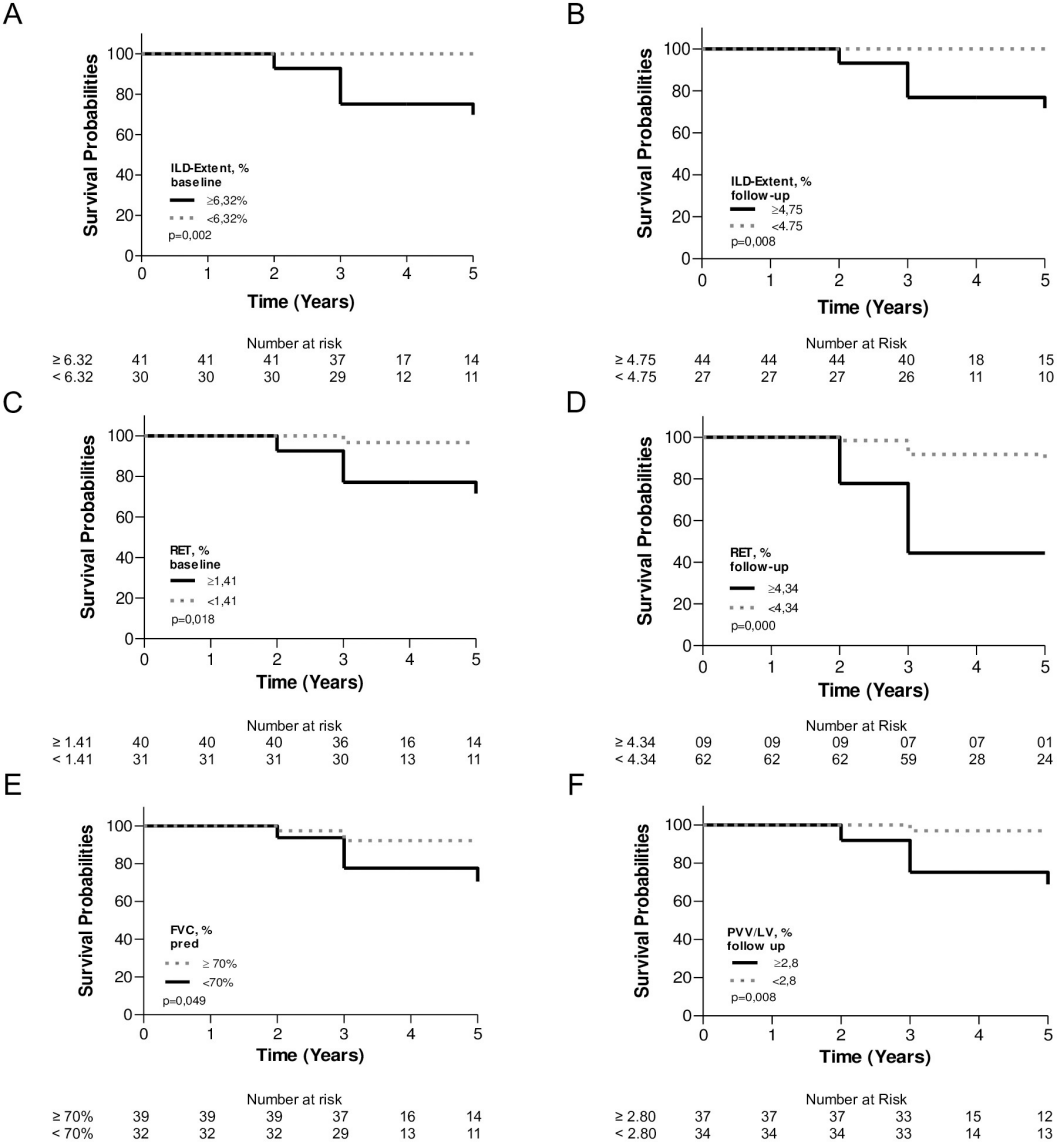
Survival compared between subgroups of patients with (A) ILD extent levels ≥ or < 6.32%; (C) reticular (RET) pattern ≥ or < 1.41% and (E) FVC ≥ or < 70% at baseline. Survival compared between subgroups of patients with (B) ILD extent ≥ or < 4.75%; (D) RET pattern ≥ or < 4.34% and (F) PVV/LV% ≥ or < 2.8% at follow-up. Survival rates in patients with ILD extension of ≥ 6.32% at baseline was 100%, 75% and 69% at 1, 3 and 5 years and of 100% at 1, 3 and 5 years in patients with ILD extension < 6.32% (A). At follow-up, the survival rates in patients with ILD extension of ≥ 4.75 was 100%, 93% and 71% at 1, 3 and 5 years and of 100% at 1, 3 and 5 years in patients with ILD extension of < 4.75 (B). Patients with a RET pattern ≥ 1.41% at baseline had survival of 100%, 77% and 71% at 1, 3 and 5 years and of 100%,96% and 96% at 1, 3 and 5 years in patients with RET < 1.41% (C). In patients with a RET pattern of ≥ 4.34 at follow-up the survival rates were 100%, 44% and 44% at 1, 3 and 5 years and of 100%, 91% and 87% at 1, 3 and 5 years in patients with RET pattern of < 4.34 (D). In patients with a FVC ≥ 70% at baseline the survival at 1,3 and 5 years was 100, 92 and 92% and of 100%, 77% and 70% in those with FVC < 70% (E). At follow-up, the survival rates in patients with PVV/LV ≥ 2.8 was 100%, 75% and 68% at 1, 3 and 5 years and of 100%, 96% and 96% at 1, 3 and 5 years in those with a PVV/LV <2.8 (F).

As shown by Kaplan‒Meier survival curves (Fig 2) disease extent ≥ 6.32% at baseline (p = 0.002) and ≥ 4.75% at follow-up (p = 0.008), as well as reticular pattern ≥ 1.41% at baseline (p = 0.018) and ≥ 4.34% at follow-up (p = 0.000), were significantly associated with higher mortality rates. Overall survival rates at 5 years were 69% for those with an ILD extent ≥ 6.32% and 71% for those with a reticular pattern ≥ 1.41% at baseline compared to 100% for patients with an ILD extent < 6.32% and 96% for those with a reticular pattern < 1.41%. FVC < 70% pred at baseline (p = 0.049) and PVV/LV ≥ 2.80 (p = 0.008) at 24 months were also significantly associated with higher mortality rates. Detailed survival rates are depicted in Fig 2.

By univariate analysis of the 71 patients, the risk factors associated with death were FVC < 70% pred at baseline (HR = 4.88; p = 0.043) and an FVC decline ≥ 5% or ≥ 10% at follow-up (HR = 7.95; p = 0.008; HR = 3.4; p = 0.041, respectively). Regarding qTC values at baseline, we observed a significantly higher risk of death among patients with a reticular pattern ≥ 1.41%, ground glass opacities ≥ 4.83% and PVV ≥ 112.18 cm^3^ (HR = 8.13; p = 0.046; HR = 9.19; p = 0.034; HR = 3.61; p = 0.041, respectively). At follow-up, qTC analysis showed a significantly higher risk of mortality among patients with a reticular pattern ≥ 4.34%, PVV ≥ 157.88 cm^3^ and PVV/LV ≥ 2.80% (HR = 7.23; p = 0.001; HR = 5.33; p = 0.006 and HR = 9.85; p = 0.029, respectively) (Table 4).

**Table 4:** Univariate analysis of clinical, functional and anatomical characteristics and risk of death.

Variables	HR	CI 95%	p
Age <52 years	2.5	0.73–8.55	0.144
Cutaneous subset diffuse	3.39	0.97–11.79	0.054
Female sex	2.05	0.44–9.55	0.359
Positive antitopoisomerase I antibodies	1.01	0.26–3.91	0.988
FVC decline ≥ 10%	3.4	1.05–11.32	0.041
FVC decline ≥5%	7.95	1.71–36.86	0.008
FVC <70 versus ≥ 70% baseline	4.88	1.05–22.62	0.043
**CALIPER**			
Reticular Pattern ≥ 1.41% baseline	8.13	1.04–63.51	0.046
Reticular Pattern ≥ 4.34% follow-up	7.23	2.17–24.08	0.001
ILD-extent ≥ 6.32% baseline	54.70	0.41–7222	0.108
ILD-extent ≥ 4.75% follow-up	42.83	0.414–6860	0.147
Ground-glass ≥ 4.83% baseline	9.19	1.17–71.84	0.034
Ground-glass ≥ 4.28% follow-up	47.44	0.32–6834	0.128
PVV ≥ 112.18 cm3 baseline	3.61	1.05–12.34	0.041
PVV ≥ 157.88 cm3 follow-up	5.33	1.61–17.56	0.006
PVV/LV ≥ 2.39% baseline	2.81	0.60–13.03	0.187
PVV/LV ≥ 2.80% follow-up	9.85	1.26–76.98	0.029

CI: confidence interval, FVC: forced vital capacity, HR: Hazard ratio, ILD: interstitial lung disease, PVV: pulmonary vessel volume, PVV/LV, %: pulmonary vessel volume per lung volume.

We used several multivariate analysis models for mortality prediction, which are described in Table 5 and in S5 and S6 Tables. The independent predictors of mortality were age (Exp 0.906, 95%CI 0.826–0.995), FVC decline ≥ 5% at follow-up (Exp 15.01, 95%CI 1.90–118.5), and a higher reticular pattern (%) on qCT at baseline (Exp 2.70, 95%CI 1.26–5.82), with the last variable showing statistical significance in all models.

**Table 5:** Multivariate assessment between sex, age, ground-glass (%) and reticular pattern (%) at baseline and FVC decline ≥5% pred at 24 months in predicting mortality.

	Exp (B)	CI 95%	p
Female sex	3.71	0.09–101.3	0.514
Age	0.906	0.826–0.995	0.038
Baseline ground-glass, %	0.904	0.804–1.02	0.091
Baseline reticular pattern, %	2.706	1.26–5.823	0.010
FVC decline ≥5%	15.01	1.90–118.5	0.010

AIC = 47, p <0.001.

CI: confidence interval, FVC: forced vital capacity

## Discussion

Due to the potential severity of ILD in SSc patients, reliable tools that enable risk stratification of progression and prognosis are extremely important in clinical practice but remain an unmet need [6, 8, 10]. In our study, we showed for the first time that quantitative HRCT assessments using CALIPER, particularly the extent of the reticular pattern at baseline, were independently associated with 5-year mortality. Furthermore, we have identified that a small extent of involvement, such as a reticular pattern ≥ 1.41% was associated with higher risk of death and may represent a window of therapeutic opportunity, before the decline in pulmonary function occurs in patients with SSc-ILD. In addition, younger age and a decline in FVC ≥ 5% pred at 24 months of follow-up were also associated with a higher risk of mortality.

Assessments of disease extent on HRCT and longitudinal declines in PFT results have been used for decades to classify clinically significant ILD [8, 10]. Goh et al. proposed an algorithm in which SSc patients are classified as having limited or extensive ILD based on the extent of lung involvement on HRCT and FVC. Patients with a fibrosis extent greater than 20% on HRCT have been shown to have a higher risk of mortality [11, 12]. Nonetheless, the assessment of the extent on HRCT is performed using visual scoring only in five levels of the lung, excluding the lung bases, which are usually involved in patients with early or limited ILD [11]. In our study, we compared the semiquantitative Goh’s score with the qCT and as expected, patients with extensive disease had higher disease extent quantified using CALIPER. The median ILD extent using qCT was of 7.82% at baseline, indicating that most patients had a low extent of the disease. Thus, in these patients, methods such as CALIPER that allow the quantification of the entire lung might be more sensitive to the early or limited detection of ILD compared with the staging system of Goh *et al*. [11, 12].

The use of qCT has grown in recent years, as it has several advantages compared to conventional HRCT. qCT has shown better reproducibility, can reduce the time required for image evaluation and can quantify the extent of the disease in the whole lung with high precision [21]. In addition, the qCT has the advantage of assessing different parenchymal patterns, including ground glass opacities, PVV, reticular pattern and honeycombing. Indeed, reticular pattern, honeycombing or PVV have been associated with mortality in patients with IPF, hypersensitivity pneumonitis and fibrotic lung disease related to autoimmune rheumatic diseases in both semiquantitative and quantitative assessments [26, 37–40].

In the present investigation, the baseline reticular pattern was associated with a higher risk of mortality by multivariate analysis. The analysis included all 71 patients, thus including patients with subclinical ILD with less than 5% of involvement on HRCT. The survival rate at 5 years was 71% in patients with a reticular pattern ≥ 1.41% compared with 96% in patients with a reticular pattern < 1.41%. Already at 3 years, the survival rates among patients with a reticular pattern ≥ 1.41% at baseline were markedly worse than those among patients with a lower percentage of the reticular pattern (77% versus 96%, respectively), indicating that the extent of the reticular pattern might be a useful tool to predict early mortality in patients with SSc. The reticular pattern is an anatomical distortion extending from the structures in the center of the lobe to the interlobular septa resulting from a previous cellular infiltrate. Thus, high expression of this pattern will lead to impairment of gas exchange and, ultimately, a decrease in pulmonary function [41]. Interestingly, a study by Jacob et al. evaluating patients with hypersensitivity pneumonitis showed that the reticular pattern analyzed using qCT and DLco was the only predictor of mortality by multivariate analysis [39].

In addition, Volkman et al. evaluated the association between quantitative ILD extent in CT images in the Scleroderma Lung Studies I and II and found that a greater than or equal to 2% change in quantification ILD (QILD) was associated with worse long-term mortality [42].

Other parameters in our work, including ground glass opacities ≥ 4.83%, PVV ≥ 112.18 cm^3^ at baseline, PVV ≥ 157.88 cm^3^ and PVV/LV ≥ 2.80% at follow-up, were also associated with a higher risk of death by univariate analysis but not by multivariate analysis. Ferrazza et al. explored CALIPER in SSc and observed that a ground glass opacity score ≥ 4.5% at baseline led to a 10% decline in DLco at 12 months, suggesting that this parameter is related to PFT worsening during follow-up [31].

In our study, ground glass changes showed the greatest percentages of extent on qCT evaluations, with a median of 6.87%. Occhipinti et al. also showed that ground glass opacities were the parameter with the greatest extent in quantified readings, although their average value was much higher than our finding (mean 18.8%) [30]. Unexpectedly, in our study, most of the qCT analyzes did not show significant differences at 24 months of follow-up. Despite a lack of statistical power, small declines were observed in ground glass opacities and reticular patterns from 6.87% [1.81–15.8] to 6.55% [1.67–19.5] and from 1.58% [0.67–2.85] to 1.43% [0.67–3.19], respectively, with an average decline of 3.93% ± 8.92% in FVC. The absence of significant changes on qCT may be explained by some characteristics of our population, including patients without ILD, a long disease duration, mostly limited SSc, and perhaps less severe ILD. Moreover, the small decrease in lung volume, could have made the qCT changes less detectable. Most patients had also received immunosuppressive treatment, and we cannot exclude the potential impact of such treatment on our results.

We found a strong correlation of quantified results with PFT measurements both at baseline and at follow-up, which is consistent with previous studies [26, 29, 32, 39]. The FVC correlated strongly with all qCT parameters, except honeycombing. The lack of correlation with honeycombing can be justified by the low percentage of this abnormality in the sample. These results are in line with expectations for a patient with SSc-ILD whose pathophysiology creates a predisposition to the presence of larger areas of ground glass opacities and a reticular pattern without honeycombing.

Using Kaplan‒Meier curves, mortality was higher in patients with extents ≥ 6.32% and reticular patterns ≥1.41%. Interestingly, a 5% disease extent was previously explored in another study with SSc patients, suggesting that this threshold could be a future cutoff point for disease stratification in SSc-ILD [32]. In our study, we also evaluated PVV and PVV/LV, which have been proposed as biomarkers of severity in SSc-ILD and IPF [28–30]. Nevertheless, in our study, PVV/LV was not associated with mortality at baseline but was strongly associated with mortality on univariate analysis at follow-up (HR = 9.85; p = 0.029).

In addition, an FVC decline ≥ 5% at 24 months was also an independent predictor of mortality by multivariate analysis, reinforcing the importance of serial PFTs for monitoring patients with ILD-SSc. Although an OMERACT Connective Tissue Disease-associated ILD working Group considered an isolated decline ≥ 10% in the predicted FVC at 24 months for disease progression [16], more recently, the ATS/ERS/JRS/ALAT guidelines considered an absolute decline in FVC ≥5% pred or in DLco ≥10% pred to be a criterion for progression [43]. Indeed, other works have also shown that a variation in FVC ≥ 5% pred was significantly associated with mortality. A Norwegian cohort of 228 patients with SSc-ILD followed up for a mean of 6.2 years showed a worse 10-year survival rate for patients with an FVC decline ≥ 5% of the predicted value [44]. Taken together, these findings indicate that a lower FVC decline, ex. ≥ 5%, should be considered an important surrogate marker for the severity of SSc-ILD.

As the identification of risk factors for the severity of ILD in initial assessments facilitates therapeutic decision-making, quantifying and assessing disease extent by qCT might be useful for the institution of early treatment, while a decline in FVC requires serial assessments, which could lead to diagnostic and therapeutic delays [8, 16].

Repeated HRCT is also a known concern for patients with SSc, as the radiation load can increase the risk of malignancy. Given that no significant abnormalities were found on qCT over a period of 24 months in our cohort, CT follow-ups performed at 24 months may represent an interesting time interval for estimating the stability or progression of the disease. These results require additional data, but once confirmed, they may increase safety in terms of the cumulative radiation dose.

This study has several limitations, including the retrospective design, sample size and measurement of DLco in a small proportion of the population evaluated. Prospective studies with a larger number of patients can confirm the findings presented here.

## Conclusion

In conclusion, our study showed that qCT has good correlations with pulmonary function test findings and that a higher percentage of the reticular pattern at baseline and the decline in FVC ≥ 5% pred at 24 months were independent predictors of mortality. Moreover, the findings of this work suggest that qCT is a sensitive diagnostic method for determining pulmonary abnormalities and to predict mortality in patients with SSc and could replace or be used in conjunction with FVC declines in future therapeutic trials and clinical applications.

## Supporting information

S1 Table Analysis of the ROC curve with the cut-off points for the qCT parameters and for FVC to distinguish between patients who died and did not. (DOCX)

S2 Table Quantified qCT parameters according to FVC ≥ 70% and < 70% in patients with SSc. (DOCX)

S3 Table Correlations between qCT patterns and PFTs variables. (DOCX)

S4 Table Clinical variables, FVC and qCT parameters according to patients who died and did not in baseline and follow up. (DOCX)

S5 Table Multivariate assessment between sex, age, ground-glass%, reticular pattern% and baseline FVC (quantitative variable) in predicting mortality. (DOCX)

S6 Table Multivariate assessment between sex, age, ground-glass%. reticular pattern% and FVC <70% of predicted in baseline in predicting mortality. (DOCX)
