# Supplementary material for: Quantitative chest computed tomography predicts mortality in systemic sclerosis: A longitudinal study
Source: PLoS One. 2024 Sep 27;19(9):e0310892. doi: 10.1371/journal.pone.0310892 (PMC11432915; doi:10.1371/journal.pone.0310892)
Supplement: S1 Table — (DOCX) [file pone.0310892.s001.docx]

**Supplementary Table S1**. Analysis of the ROC curve with the cut-off points for the qCT parameters and for FVC to distinguish between patients who died and did not

| Variable qCT | Cut-off | AUC  (95% CI) | p | Youden | Sensitivity (%) | Specificity  (%) |
| --- | --- | --- | --- | --- | --- | --- |
| ILD extent, % baseline | 6.32 | 0.625 (0.502 - 0.737) | 0.042 | 0.533 | 100 | 53 |
| ILD extent, % follow-up | 4.75 | 0.700 (0.580 - 0.803) | 0.003 | 0.450 | 100 | 45 |
| Reticular pattern, % baseline | 1.41 | 0.753 (0.636 - 0.848) | 0.001 | 0.442 | 90 | 53 |
| Reticular pattern, % follow-up | 4.34 | 0.724 (0.605 - 0.824) | 0.009 | 0.388 | 93 | 45 |
| Ground-glass, % baseline | 4.83 | 0.630 (0.498-0.763) | 0.054 | 0.442 | 90 | 53 |
| Ground-glass, % follow-up | 4.28 | 0.697 (0.567-0.827) | 0.003 | 0.466 | 100 | 46 |
| PVV, baseline | 112.1 | 0.645 (0.428-0.863) | 0.189 | 0.369 | 63 | 73 |
| PVV, follow-up | 157.8 | 0.662 (0.452-0.872) | 0.130 | 0.371 | 91 | 45 |
| PVV/LV, % baseline | 2.39 | 0.642 (0.476-0.809) | 0.092 | 0.252 | 81 | 43 |
| PVV/LV, % follow up | 2.80 | 0.683 (0.562 – 0.789) | 0.032 | 0.368 | 81 | 55 |
| FVC, % (baseline) | 70 | 0.664 (0.542 -0.771) | 0.034 | 0.444 | 72 | 71 |

AUC: Area Under the Curve, CI: confidence interval, FVC: forced vital capacity, ILD: Interstitial lung disease; ILD extent, %= (Ground-glass opacities, % + reticular pattern, % + Honeycombing, %), PVV: pulmonary vessel volume, PVV/LV, %: pulmonary vessel volume per lung volume.
