# Supplementary material for: Quantitative chest computed tomography predicts mortality in systemic sclerosis: A longitudinal study
Source: PLoS One. 2024 Sep 27;19(9):e0310892. doi: 10.1371/journal.pone.0310892 (PMC11432915; doi:10.1371/journal.pone.0310892)
Supplement: S2 Table — (DOCX) [file pone.0310892.s002.docx]

**Supplementary Table S2.** Quantified qCT parameters according to FVC ≥ 70% and < 70% in patients with SSc

| BASELINE | FVC < 70% | FVC ≥ 70% | p |
| --- | --- | --- | --- |
| Normal, % | 80.45 [72.0-87.1] | 94.50 [88.6-98.4] | <0.001 |
| Fibrosis score, % | 2.68 [1.69-4.78] | 0.89 [0.38-1.86] | <0.001 |
| ILD-Extent, % | 18.37 [12.0-24.0] | 4.57[1.12-6.46] | <0.001 |
| Ground-glass, % | 15.70 [8.38-21.0] | 2.92 [0.69-5.29] | <0.001 |
| Reticular pattern, % | 2.37 [1.62-4.73] | 0.88 [0.34-1.74] | <0.001 |
| Honeycombing, % | 0.023 [0.008-0.08] | 0.013 [0.007-0.03] | 0.119 |
| PVV, cm3 | 110.34 [85.4-133] | 84.75 [69.4-112] | 0.003 |
| PVV/LV, % | 3.98 [3.39-4.67] | 2.28[1.74-2.75] | <0.001 |
| FOLLOW UP |  |  |  |
| Normal, % | 78.72 [64.7-88.5] | 92.40 [86.7-98.0] | <0.001 |
| Fibrosis score, % | 2.09 [1.07-4.50] | 0.821 [0.378-2.09] | <0.001 |
| ILD-Extent, % | 17.25 [10.0-34.8] | 2.95 [1.08-7.96] | <0.001 |
| Ground-glass, % | 13.03 [6.69-28.4] | 1.85 [0.69-5.70] | <0.001 |
| Reticular pattern, % | 2.0 [1.07-3.99] | 0.80 [0.32-1.97] | <0.001 |
| Honeycombing, % | 0.021[0.0052-0.06] | 0.014[0.0052-0.03] | 0.534 |
| PVV, cm3 | 118.07 [94.0-158] | 78.64 [67.7-92] | 0.002 |
| PVV/LV, % | 4.50 [3.08-5.07] | 2.06 [1.76-2.79] | 0.002 |

NOTE: FVC = (forced vital capacity), PVV: pulmonary vessel volum, PVV/LV, %: pulmonary vessel volum per lung volum,
