# Supplementary material for: Quantitative chest computed tomography predicts mortality in systemic sclerosis: A longitudinal study
Source: PLoS One. 2024 Sep 27;19(9):e0310892. doi: 10.1371/journal.pone.0310892 (PMC11432915; doi:10.1371/journal.pone.0310892)
Supplement: S3 Table — (DOCX) [file pone.0310892.s003.docx]

**Supplementary Table S3.** Correlations between qCT patterns and PFTs variables

|  |  | Baseline | | Follow-up | |
| --- | --- | --- | --- | --- | --- |
|  |  | FVC1 | DLCO 1 | FVC2 | DLCO2 |
| Normal, % | Rho | 0.613 | 0.810 | 0.507 | 0.733 |
|  | p | < .001 | 0.022 | < .001 | 0.031 |
| ILD-Extent, % | Rho | -0.662 | -0.714 | -0.638 | -0.700 |
|  | p | < .001 | 0.058 | < .001 | 0.043 |
| Fibrosis score, % | Rho | -0.565 | -0.810 | -0.551 | -0.633 |
|  | p | < .001 | 0.022 | < .001 | 0.076 |
| Ground-glass, % | Rho | -0.619 | -0.714 | -0.639 | -0.650 |
|  | p | < .001 | 0.058 | < .001 | 0.067 |
| Reticular pattern, % | Rho | -0.564 | -0.810 | -0.568 | -0.667 |
|  | p | < .001 | 0.022 | < .001 | 0.059 |
| Honeycombing, % | Rho | -0.179 | -0.310 | -0.111 | -0.450 |
|  | p | 0.136 | 0.462 | 0.356 | 0.230 |
| PVV/LV, % | Rho | -0.661 | -0.881 | -0.697 | -0.683 |
|  | p | < .001 | 0.007 | < .001 | 0.050 |
| PVV, cm3 | Rho | -0.370 | -0.595 | -0.470 | -0.733 |
|  | p | 0.001 | 0.132 | < .001 | 0.031 |

NOTE: DLco, %= (carbon monoxide diffusing capacity), FVC = (forced vital capacity), PVV: pulmonary vessel volum, PVV/LV, %: pulmonary vessel volum per lung volum, Rho= Spearman correlation coefficient;
