# Supplementary material for: Quantitative chest computed tomography predicts mortality in systemic sclerosis: A longitudinal study
Source: PLoS One. 2024 Sep 27;19(9):e0310892. doi: 10.1371/journal.pone.0310892 (PMC11432915; doi:10.1371/journal.pone.0310892)
Supplement: S4 Table — (DOCX) [file pone.0310892.s004.docx]

**Supplementary Table S4.** Clinical variables, FVC and qCT parameters according to patients who died and did not in baseline and follow up

|  | Yes (n=11) | No (n=60) | p |
| --- | --- | --- | --- |
| Age (mean ± SD) | 48.9 ± 12.8 | 55.2 ± 11.2 | 0.090 |
| Female sex, n (%) | 9 (81.8%) | 55 (91.6%) | 0.325 |
| Disease duration (mean ± SD) | 6.55 ± 1.97 | 12.2 ± 6.60 | 0.006 |
| Diffuse cutaneous SSc, n (%) | 7 (63.6%) | 19 (31.6%) | 0.046 |
| BASELINE |  |  |  |
| FVC % pred (mean ± SD) | 62.4 ± 15.2 | 75.6 ± 19.1 | 0.033 |
| Normal, % | 85.08 [76.8-90.8] | 90.79 [79.2-96.6] | 0.160 |
| Fibrosis score, % | 3.87 [1.78-5.18] | 1.45 [0.57-2.58] | 0.009 |
| ILD-extent, % | 13.8 [8.57-17.0] | 6.18 [2.35-19.6] | 0.090 |
| Ground-glass, % | 9.81 [6.76-14.3] | 4.51 [1.43-15.8] | 0.174 |
| Reticular pattern, % | 3.14 [1.77-5.14] | 1.43 [0.5-2.48] | 0.008 |
| Honeycombing, % | 0.013 [0.004-0.06] | 0.016 [0.01-0.05] | 0.769 |
| PVV, cm3 | 126.59 [75.7-160] | 92.74 [76.7-119] | 0.129 |
| PVV/LV, % | 3.74 [2.55-4.38] | 2.67 [2.05-4.07] | 0.056 |
| FOLLOW UP |  |  |  |
| FVC % pred (mean ± SD) | 52.0 ± 14.8 | 72.9 ± 17.8 | <.001 |
| Normal, % | 79.3 [70.1-89.2] | 89.3 [78.1-95.6] | 0.155 |
| Fibrosis score, % | 3.36 [1.17-5.14] | 1.28 [0.6-2.6] | 0.026 |
| ILD-extent, % | 14.0 [10.8-25.3] | 7.0 [2.12-16.2] | 0.037 |
| Ground-glass, % | 12.6 [7.57-21.2] | 5.18 [1.27-12.6] | 0.040 |
| Reticular pattern, % | 3.32 [1.15-5.03] | 1.21 [0.54-2.45] | 0.019 |
| Honeycombing, % | 0.029 [0.014-0.04] | 0.015 [0.00-0.03] | 0.174 |
| PVV, cm3 | 132 [77.3-168] | 91.6 [71.8-123] | 0.091 |
| PVV/LV, % | 4.13 [2.96-5.06] | 2.73 [1.88-4.2] | 0.056 |

NOTE: FVC = (forced vital capacity), PVV: pulmonary vessel volum, PVV/LV, %: pulmonary vessel volum per lung volum,
