# Supplementary material for: Quantitative chest computed tomography predicts mortality in systemic sclerosis: A longitudinal study
Source: PLoS One. 2024 Sep 27;19(9):e0310892. doi: 10.1371/journal.pone.0310892 (PMC11432915; doi:10.1371/journal.pone.0310892)
Supplement: S5 Table — (DOCX) [file pone.0310892.s005.docx]

**Supplementary Table S5.** Multivariate assessment between sex, age, ground-glass%, reticular pattern% and baseline FVC (quantitative variable) in predicting mortality

|  | Exp (B) | CI 95% | p |
| --- | --- | --- | --- |
| Female sex | 2.99 | 0.08-106.46 | 0.548 |
| Age | 0.942 | 0.872-1.01 | 0.125 |
| Ground-glass, % | 0.911 | 0.827-1.03 | 0.057 |
| Reticular pattern, % | 2.389 | 1.17-4.87 | 0.017 |
| FVC baseline | 0.982 | 0.827-1.00 | 0.497 |

Note: AIC= 56, CI: confidence interval, p=0.0005.
