# Supplementary material for: Quantitative chest computed tomography predicts mortality in systemic sclerosis: A longitudinal study
Source: PLoS One. 2024 Sep 27;19(9):e0310892. doi: 10.1371/journal.pone.0310892 (PMC11432915; doi:10.1371/journal.pone.0310892)
Supplement: S6 Table — (DOCX) [file pone.0310892.s006.docx]

**Supplementary Table S6.** Multivariate assessment between sex, age, ground-glass%. reticular pattern% and FVC <70% of predicted in baseline in predicting mortality

|  | Exp (B) | CI 95% | p |
| --- | --- | --- | --- |
| Female sex | 2.663 | 0.082-86.21 | 0.581 |
| Age | 0.938 | 0.869-1.012 | 0.096 |
| Ground-glass, % | 0.912 | 0.828-1.005 | 0.063 |
| Reticular pattern, % | 2.194 | 1.139-4.227 | 0.019 |
| FVC < 70%, pred | 1.67 | 0.268-10.52 | 0.580 |

Note: AIC=56, CI: confidence interval, p=0.005.
